# MicroRNAs Are Indispensable for Reprogramming Mouse Embryonic Fibroblasts into Induced Stem Cell-Like Cells

**DOI:** 10.1371/journal.pone.0039239

**Published:** 2012-06-21

**Authors:** Byeong-Moo Kim, Marc-Christian Thier, Sangnam Oh, Richard Sherwood, Chryssa Kanellopoulou, Frank Edenhofer, Michael Y. Choi

**Affiliations:** 1 Department of Medicine/GI Unit, Massachusetts General Hospital, Boston, Massachusetts, United States of America; 2 Harvard Medical School, Boston, Massachusetts, United States of America; 3 Institute of Reconstructive Neurobiology, Life and Brain Center, University of Bonn, Bonn, Germany; 4 Brigham and Women’s Hospital, Boston, Massachusetts, United States of America; 5 Laboratory of Immunology, National Institute of Allergy and Infectious Diseases, National Institutes of Health, Bethesda, Maryland, United States of America; Mayo Clinic, United States of America

## Abstract

MicroRNAs play a pivotal role in cellular maintenance, proliferation, and differentiation. They have also been implicated to play a key role in disease pathogenesis, and more recently, cellular reprogramming. Certain microRNA clusters can enhance or even directly induce reprogramming, while repressing key proteins involved in microRNA processing decreases reprogramming efficiency. Although microRNAs clearly play important roles in cellular reprogramming, it remains unknown whether microRNAs are absolutely necessary. We endeavored to answer this fundamental question by attempting to reprogram *Dicer*-null mouse embryonic fibroblasts (MEFs) that lack almost all functional microRNAs using a defined set of transcription factors. Transduction of reprogramming factors using either lentiviral or piggyBac transposon vector into two, independently derived lines of *Dicer*-null MEFs failed to produce cells resembling embryonic stem cells (ESCs). However, expression of human *Dicer* in the *Dicer*-null MEFs restored their reprogramming potential. Our study demonstrates for the first time that microRNAs are indispensable for dedifferentiation reprogramming.

## Introduction

MicroRNAs (miRNAs) have emerged as a new category of genes that influence many cellular processes including proliferation and differentiation. miRNAs are small, noncoding, single stranded RNAs usually 22 nucleotides long that can base pair with target mRNAs in the open reading frame or 3′ untranslated region [1]. miRNAs downregulate target genes by inhibiting protein translation and destabilizing mRNAs via deadenylation [2]. To generate functional miRNAs along the canonical pathway, two serial RNA cleavage steps involving two RNase III-containing enzymes are necessary. First, the Microprocessor complex formed by the hairpin recognizing RNA binding protein, Dgcr8, and the RNase III enzyme, Drosha, cleaves the primary miRNA transcript (pri-miRNAs) to form precursor miRNAs (pre-miRNAs) in the nucleus [3], [4], [5], [6]. Next, Exportin-5 transports the pre-miRNA to the cytoplasm [7], [8] where, a second RNase III-containing enzyme, Dicer, cleaves it to generate mature miRNAs in the cytoplasm [9], [10]. After Dicer cleavage, the gene-silencing, guide strand is able to associate with miRNA-induced silencing complex, which assists in the contact between the miRNA and the target mRNA [11]. In addition to the canonical pathway, Dicer processes all miRNAs along the non-canonical biogenesis pathways except in a few rare examples [12], [13], [14], [15], and thus, without Dicer, the cell lacks almost all mature miRNAs. Finally, Dicer has been found to process endogenous small interfering RNAs (siRNAs) in oocytes and ESCs [16], [17], [18]. However, whether endogenous siRNAs function or even exist in mammalian somatic cells including MEFs remains unknown [19].

Highlighting the importance of these pathways, targeted deletion of the *Dicer* gene in mice causes embryonic lethality at embryonic day (E) 7.5 [20], [21]. In fact, the embryos probably start to arrest at a stage prior to E7.5 because the number of *Dicer*-null embryos is about 50% lower than expected from Mendelian ratios. Mice that lack Dicer may survive to E7.5 because of the presence of maternal Dicer protein in the cytoplasm [20]. Despite early embryonic lethality in these mutant mice, at least two research groups have been able to generate *Dicer*-null ESC lines [22], [23]. Remarkably, these mutant mouse ESCs are viable and retain typical morphology of wild-type ESCs, forming oval-shaped colonies. They also express ESC specific markers, including *Oct4*, at levels comparable to wild-type ESCs. However, as compared to wild-type, *Dicer*-deficient ESCs proliferate much more slowly and do not exhibit pluripotent differentiation capability.

Similar to transcription factors, miRNAs have the ability to modulate the expression of several genes, and therefore, contribute significantly to cellular gene expression programs. This is likely the reason why miRNAs have potent functions not only in normal cellular processes and in diseased states, but also in forced reprogramming of somatic cells into induced pluripotent stem cells (iPSCs) [24], [25], [26], [27], [28]. For instance, members of ESC-specific cell cycle-regulating miRNAs enhance the efficiency of cellular reprogramming when *Oct4*, *Sox2*, and *Klf4* are transduced, and can replace Myc in reprogramming mouse fibroblasts to iPSCs by acting downstream of Myc [27]. More recently, iPSCs were successfully generated by lentiviral expression of the *miR302/367* cluster or transient transfection of miRNA mimics, *miR200c*, and clusters of *miR302*s, and *miR369*s, without any exogenous transcription factor expression [25], [28]. Finally, repressing key miRNA processing molecules such as Drosha, Dicer, and Ago2 resulted in significant decrease in reprogramming efficiency [29]. The knockdown approach used in this study was reported to repress between 70–80% in reprogramming efficiency. Hence, although it was demonstrated that miRNAs as a whole are able to modulate reprogramming, it remains unclear whether they are in fact necessary for cellular reprogramming. In this study, we formally answer this question by attempting to reprogram *Dicer*-null MEFs that lack almost all functional miRNAs by transducing a set of defined transcription factors known to activate the dedifferentiation program. Although two different gene delivery methods were used on two independently derived *Dicer*-null MEFs, combinations of *Oct4*, *Sox2*, *Klf4*, *cMyc*, and *Lin28* failed to generate cells that resemble *Dicer*-null ESCs. However, *Dicer*-null, induced stem cell-like cells were successfully produced when the human *Dicer* homologue was introduced in *Dicer*-null MEFs before the dedifferentiation step, suggesting that miRNAs are indispensable for cellular reprogramming.

## Results and Discussion

### *Dicer^Δ/Δ^* Mouse Embryonic Fibroblasts Lacking miRNAs are Viable Despite Suppressed Proliferation

**Figure 1:**
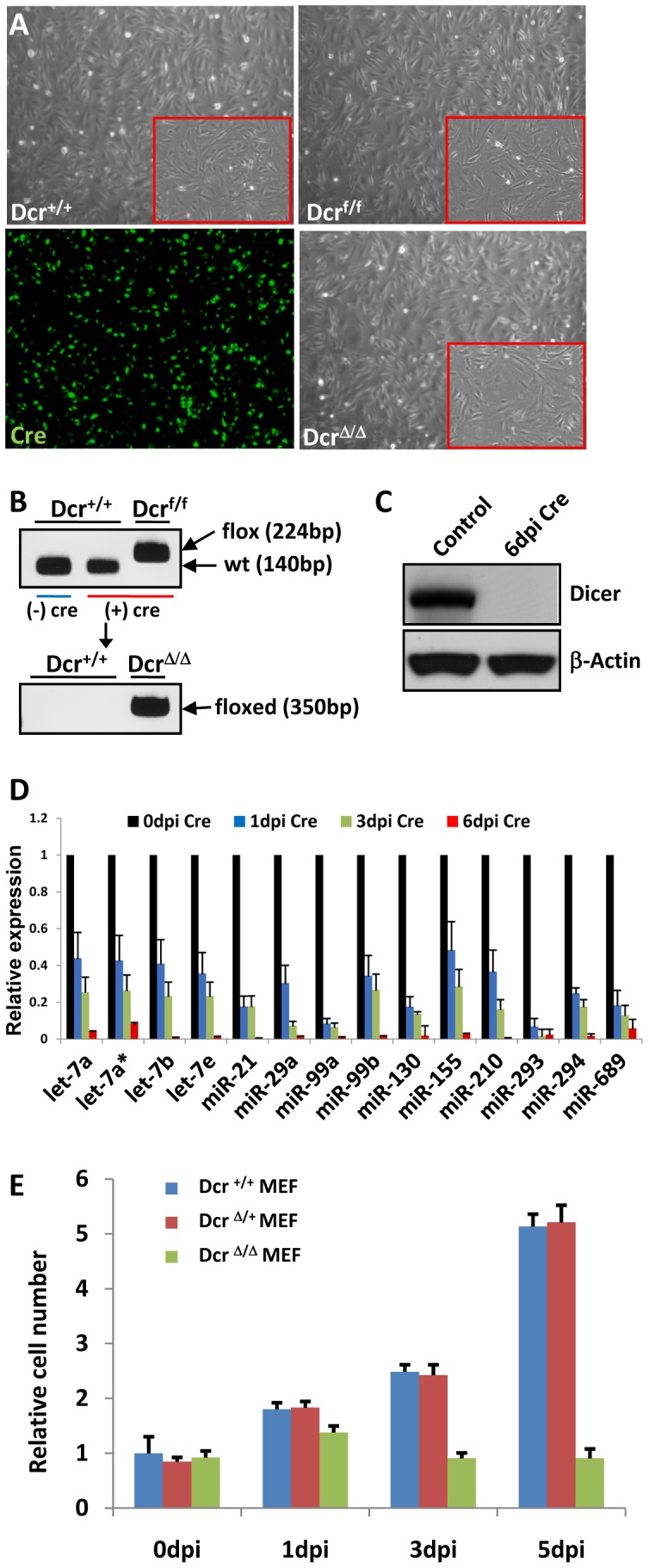
*Dicer*-null MEFs are viable despite suppressed proliferation. (**A**) Adeno-Cre virus was able to infect MEFs at high efficiency, judged by co-expression of GFP signal. *Dicer* ^Δ/Δ^ MEF generated from *Dicer* ^f/f^ MEF by Cre excision of *Dicer* gene had typical cellular morphology similar to wild-type (*Dicer* ^+/+^) MEF. Boxed areas represent magnified view. (**B**) PCR analysis of genomic DNA to demonstrate Cre excision of *Dicer* gene to generate *Dicer* ^Δ/Δ^ (floxed DNA band) MEF from *Dicer* ^f/f^ MEF (flox DNA band). (**C**) Dicer immunoblot confirmed that Cre induction led to deletion of *Dicer* gene and prevention of Dicer protein expression. By 6 days post induction (dpi) with Cre, Dicer protein was completely depleted. (**D**) qRT-PCR of select miRNAs confirmed reduction in the levels of mature forms of miRNA in *Dicer* ^Δ/Δ^ MEFs. The levels of most mature miRNAs tested were almost completely depleted by 6 days after Cre induction. Each value is represented relative to an assigned 0 dpi value of 1.0 for that miRNA. Data are presented as mean +/− SD. (**E**) *Dicer* ^Δ/Δ^ MEFs demonstrated a proliferation delay. In contrast, *Dicer* ^Δ/+^ MEFs retained normal proliferation rate resembling *Dicer* ^+/+^ MEFs. All values are represented relative to an assigned *Dicer* ^+/+^ MEF value of 1.0 at 0 dpi. Data are presented as mean +/− SD.

To test whether miRNAs are necessary for reprogramming a somatic cell type into induced stem cell-like cells, we first generated MEFs that lack almost all miRNAs. We utilized two different *Dicer*-null MEF lines from two independently generated mutant mouse lines that have different *Dicer* exons flanked by *loxP* sites [22], [30]. When we crossed *Dicer* ^f/+^ mice, resulting pups were *Dicer* ^+/+^, *Dicer* ^f/+^, and *Dicer* ^f/f^ in 1∶2:1 ratio. *Dicer* ^f/f^ MEFs harvested from E13.5 embryos proliferated normally and had morphology resembling wild-type MEFs (Fig 1A). However, once Cre recombinase was delivered by infecting cells with recombinant adenovirus (Adeno) encoding Cre, *Dicer* ^f/f^ MEFs lost both functional *Dicer* alleles to become *Dicer* ^Δ/Δ^ (*Dicer*-null) MEFs (Fig 1A, B). Adeno-Cre virus was able to consistently infect greater than 90% of MEFs at a multiplicity of infection (MOI) of ∼100, judged by co-expression of a GFP reporter (Fig 1A). To confirm that infection with Adeno-Cre virus led to deletion of the *Dicer* gene and prevention of Dicer protein expression, we performed immunoblot for Dicer protein. By 6 days post-induction (dpi) with Cre, Dicer protein was completely depleted (Fig 1C). We also confirmed reduction in the levels of select mature miRNAs in *Dicer* ^Δ/Δ^ MEFs. Reverse transcription-quantitative polymerase chain reaction (qRT-PCR) revealed that the levels of most mature miRNAs tested were nearly 98% depleted by 6 dpi. (Fig 1D). *Dicer*-null MEFs had a typical cellular morphology comparable to wild-type MEFs. However, *Dicer*-null MEFs demonstrated a proliferation delay, while *Dicer* ^Δ/+^ MEFs retained normal proliferation rate resembling wild-type MEFs (Fig 1E). These results were in line with published phenotype of *Dicer*-null MEFs from independently generated conditional *Dicer* knockout mice [31].

### *Dicer^Δ/Δ^* Mouse Embryonic Fibroblasts Lacking miRNAs Fail to Reprogram

**Figure 2:**
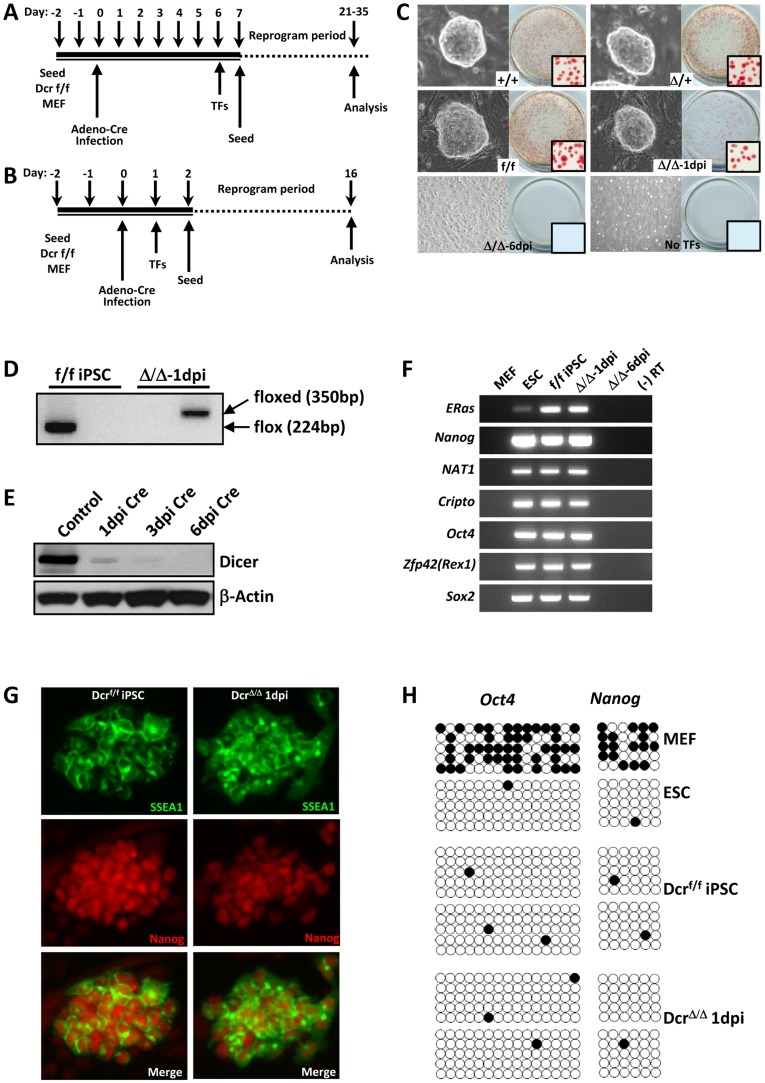
*Dicer*-null MEFs lacking miRNAs fail to reprogram. (**A, B**) Timelines of attempt at reprogramming *Dicer*-null MEFs. The main difference between the two strategies is that (**A**) transduces reprogramming transcription factors (TFs) 6 days post induction with Cre, while (**B**) transduces TFs 1 day post induction with Cre. *Dicer*-null MEFs could not be reprogrammed when reprogramming factors were transduced 6 days after induction. However, reprogramming *Dicer*-null MEFs was possible when reprogramming factors were transduced 1 day post induction with Cre. (**C**) *Dicer* ^+/+^, *Dicer* ^Δ/+^, and *Dicer* ^f/f^ MEFs consistently reprogrammed into iPSCs with reprogramming factors. These iPSCs stained for alkaline phosphatase. *Dicer* ^Δ/Δ^ MEFs reprogrammed to form induced stem cell-like cell colonies that stained for alkaline phosphatase when reprogramming factors were transduced 1 day post induction with Cre (Δ/Δ-1dpi). Transducing reprogramming factors 6 dpi (Δ/Δ-6dpi) or not transducing any factors (No TF) never reprogrammed *Dicer*-null MEFs. Boxed areas represent magnified view. (**D**) Genomic PCR confirmed induced stem cell-like cell colonies formed by transducing reprogramming factors 1 day post induction with Cre (Δ/Δ-1dpi) having *Dicer* ^Δ/Δ^ genotype (floxed DNA band). Control iPSC colonies (f/f iPSC) formed without Cre induction had *Dicer* ^f/f^ genotype (flox DNA band). (**E**) Residual Dicer protein is still present 1 day after deletion of *Dicer* gene. By 6 days post induction (dpi) with Cre, residual Dicer protein is completely degraded, inhibiting cellular reprogramming. (**F, G**) Wild-type ESCs, *Dicer* ^f/f^ iPSCs (f/f iPSC), and *Dicer* ^Δ/Δ^ induced stem cell-like cells generated by transducing reprogramming factors 1 day post induction with Cre (Δ/Δ-1dpi) expressed all stem cell markers tested by RT-PCR (**F**), and immunofluorescence (**G**). (**H**) *Dicer* ^f/f^ iPSCs and *Dicer* ^Δ/Δ^ induced stem cell-like cells acquired ESC methylation patterns in *Oct4* and *Nanog* promoters.

We attempted to reprogram *Dicer* ^Δ/Δ^ MEFs using defined sets of transcription factors known to dedifferentiate various somatic cells into iPSCs [32], [33], [34]. We used two combinations of transcription factors, *Oct4*, *Sox2*, *Klf4*, *cMyc*, and *Lin28* (5 TFs), and *Oct4*, *Sox2*, *Klf4*, and *cMyc* (4 TFs). Transducing 5 TFs increases reprogramming efficiency by twofold compared to 4 TFs [34]. In addition, we tested two different gene delivery methods to reprogram MEFs, piggyBac transposon carrying 2A peptide-linked reprogramming factors and a polycistronic doxycycline-inducible lentiviral system [34], [35]. PiggyBac transposon vector transfection efficiencies in control and *Dicer* ^Δ/Δ^ MEFs were 30–40% and 25–35% respectively. As mentioned, we also used two different MEF lines produced from two independently generated mutant mice with floxed-*Dicer* alleles [22], [30]. Despite these variations and optimizations, *Dicer* ^Δ/Δ^ MEFs could not be reprogrammed into induced stem cell-like cells when either 4 TFs or 5 TFs were transduced 6 days after induction with Cre (Fig 2A), when all mature miRNAs were depleted. We use the term “induced stem cell-like cells" instead of iPSCs because the reprogrammed cells without miRNAs would not be pluripotent. Instead, they would resemble *Dicer*-null ESCs known to have severe proliferation and differentiation defects [22], [23]. Typically, iPSC colonies appear after 2 weeks of expression with 4 TFs; since the *Dicer*-null ESCs proliferate poorly, we decided to extend our reprogramming duration longer. However, we did not detect any reprogrammed cells even after 4 weeks post transduction of defined reprogramming factors.

Meanwhile, we were able to consistently and reliably reprogram control MEFs with genotypes *Dicer* ^+/+^, *Dicer* ^Δ/+^, and *Dicer* ^f/f^ into iPSCs using either 4 TFs or 5 TFs with the overall reprogramming efficiency between 0.3% and 0.5% (Supp Table S1), in line with published reports [34], [35]. These iPSCs grew in colonies in the presence of leukemia inhibitory factor, stained for alkaline phosphatase (Fig 2C), expressed all stem cell markers tested (Fig 2F, G), acquired stem cell methylation pattern in *Oct4* and *Nanog* promoters (Fig 2H), and differentiated normally to all three germ layers during teratoma assays (Data not shown). Control MEFs required 2 weeks of culturing after delivering 4 TFs or 5 TFs to generate iPSCs, consistent with published results [34], [35].

### *Dicer^Δ/Δ^* Mouse Embryonic Fibroblasts that Still have Residual miRNAs can Reprogram

Although it was clearly evident that MEFs lacking miRNAs could not be reprogrammed we identified one condition in which reprogramming was possible even after Cre excision of relevant *Dicer* exons. When we transduced 4 TFs or 5 TFs one day after expressing Cre recombinase (Fig 2B), a few *Dicer* ^Δ/Δ^ colonies, confirmed by genomic PCR, formed after 2 weeks with the overall reprogramming efficiency of less than 0.3% (Fig 2C, D; Supp Table S1). These induced stem cell-like cells reprogrammed from *Dicer* ^Δ/Δ^ MEFs grew in colonies, acquired cellular morphology similar to ESCs, expanded indefinitely, and stained for alkaline phosphatase (Fig 2C). They also expressed all ESC markers tested by RT-PCR (Fig 2F) and immunofluorescence (Fig 2G), and acquired methylation patterns of *Nanog* and *Oct4* promoters that were similar to control ESC and iPSC (Fig 2H). However, there were several phenotypic features that were dissimilar to control ESCs and iPSCs. *Dicer* ^Δ/Δ^ induced stem cell-like cells proliferated slower than control ES and iPSCs. Furthermore, these cells failed to differentiate into endoderm with Activin and other growth factors in culture, a condition that regularly yields more than 95% endoderm from ESCs (Data not shown) [36]. Finally, these cells could not give rise to any recognizable teratoma with germ layers upon subcutaneous injection into severe combined immunodeficiency (SCID) mice, demonstrating their severe differentiation defect. These mutant phenotypes were reminiscent of *Dicer*-null ESCs which also had severe proliferation and differentiation defects [22]. We were able to generate induced stem cell-like cells only when the combination of 4 TFs or 5 TFs were delivered just one day after Cre induction, but not if reprogramming factors were transduced 6 days after Cre induction. We believe that reprogramming *Dicer* ^Δ/Δ^ MEFs was possible only when reprogramming factors were introduced one day after Cre induction because residual Dicer protein and mature miRNAs are still present up to 3 days after deletion of *Dicer* gene (Fig 1D, 2E). By day 6 after Cre induction, residual Dicer protein and miRNAs are almost completely absent, inhibiting cellular reprogramming (Fig 1D, 2E). Likewise, the effect of residual Dicer protein has been recognized previously *in vivo*. Residual maternal Dicer protein in the absence of *Dicer* gene may allow prolonged survival of mouse and zebrafish Dicer mutant embryos [20], [37].

### Human *Dicer* Expression in *Dicer^Δ/Δ^* Mouse Embryonic Fibroblasts Allows Generation of iPSCs

**Figure 3:**
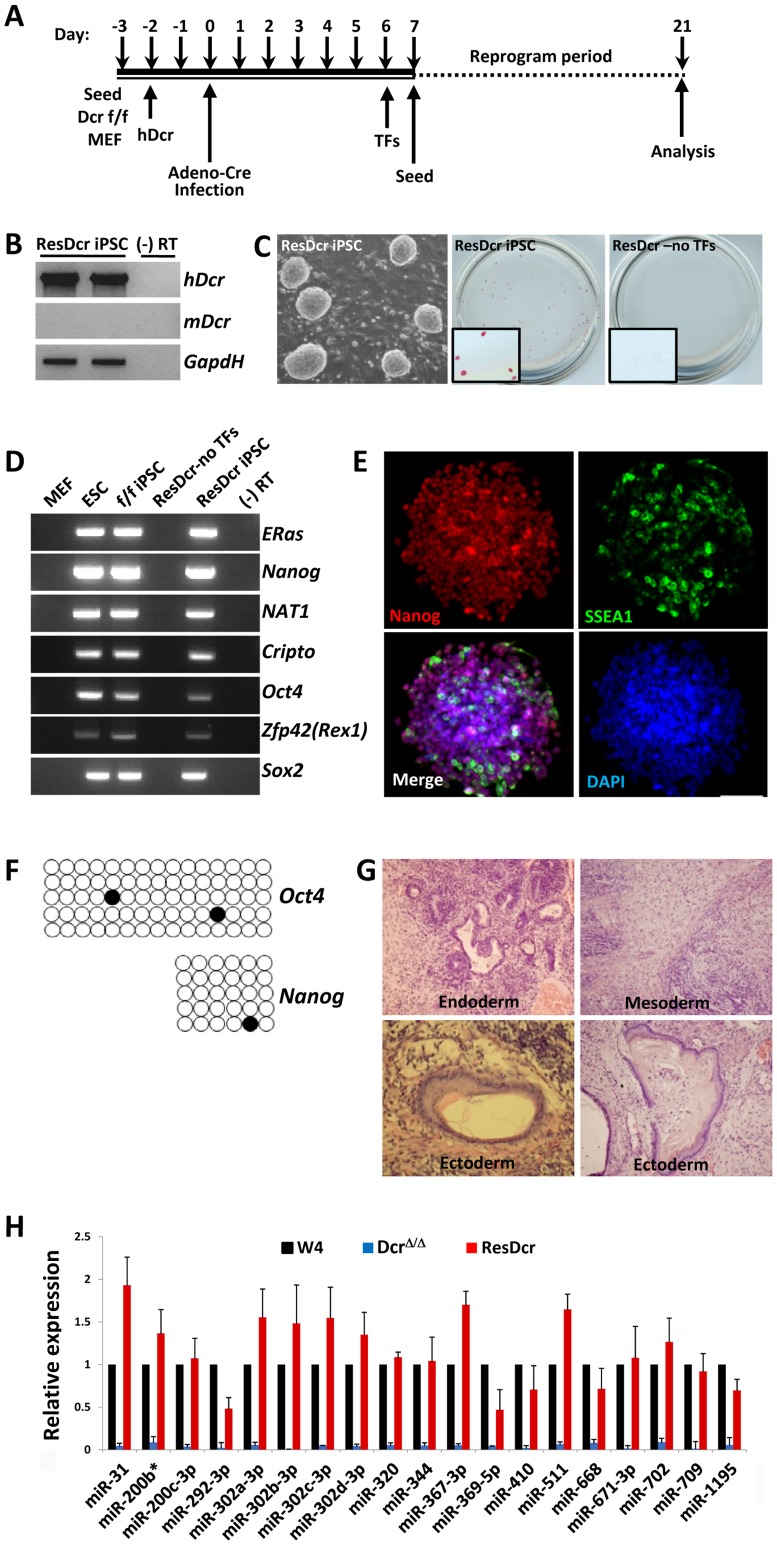
Human *Dicer* expression in *Dicer*-null MEFs allows generation of iPSCs. (**A**) Timeline of reprogramming *Dicer*-null MEFs rescued with human *Dicer*. Once human *Dicer* cDNA integrated into the *Dicer* ^Δ/Δ^ MEF genome, reprogramming became possible even when factors were transduced 6 days after Cre induction. (**B**) Rescued iPSCs (ResDcr iPSC) lacked mouse *Dicer* (*mDcr*), but instead expressed human *Dicer* (*hDcr*) gene, verified by RT-PCR. (**C, D, E**) *Dicer* ^Δ/Δ^ MEFs expressing human *Dicer* can reprogram to become iPSCs. Rescued iPSCs expressing human *Dicer* showed ESC morphology and stained for alkaline phosphatase (boxed areas represent magnified view) (**C**), and expressed stem cell markers tested by RT-PCR (**D**) and immunofluorescence (**E**). (**F**) Rescued iPSC promoters for stem cell genes *Oct4* and *Nanog* became demethylated, resembling wild-type ESCs. (**G**) Upon subcutaneous injection into SCID mice, rescued iPSCs formed teratomas that showed differentiation into all three germ layers. (**H**) Human Dicer can cleave mouse pre-miRNAs into mature miRNAs. qPCR for a panel of mature miRNAs in rescued iPSCs (ResDcr) lacking mouse *Dicer* demonstrated comparable expression levels to that of wild-type ESCs (W4). In contrast, mature miRNAs were completely depleted in *Dicer*-null ESCs (Dicer^Δ/Δ^). Each value is represented relative to an assigned W4 value of 1.0 for that miRNA. Data are presented as mean +/− SD.

To verify that the inability to reprogram *Dicer* ^Δ/Δ^ MEFs was truly due to lack of functional Dicer protein and miRNAs, and not from an unrecognized mutation or variability in our assays, we attempted to rescue the capacity to reprogram by reintroducing *Dicer* gene into *Dicer*-null MEFs. We overexpressed reprogramming factors after integrating human *Dicer* cDNA into the genome of *Dicer* ^Δ/Δ^ MEFs using a piggyBac expression vector. Human Dicer protein has 93% sequence identity with its mouse homologue, and shares the key enzymatic function through the conserved ribonuclease III C terminal domain. Even when we transduced reprogramming factors 6 days after Cre induction to delete mouse *Dicer* exons, we were able to generate iPSCs from MEFs that expressed the human *Dicer* homologue (Fig 3A). These iPSCs lacked mouse *Dicer*, but instead expressed the human *Dicer* gene, as confirmed by RT-PCR (Fig 3B). Within 2 weeks after transducing reprogramming factors, *Dicer* ^Δ/Δ^ MEFs expressing human *Dicer* dedifferentiated to become iPSCs that grew in colonies. Reprogrammed iPSCs expressing human *Dicer* displayed typical ESC morphology, stained for alkaline phosphatase, and expressed stem cell markers (Fig 3C, D, E). Their promoters for stem cell genes *Oct4* and *Nanog* became demethylated, resembling wild-type ESCs (Fig 3F). Upon subcutaneous injection into SCID mice, these cells formed teratomas that showed differentiation into all three germ layers (Fig 3G). Finally, to confirm that human Dicer has a robust enzymatic activity and can cleave mouse pre-miRNAs into mature miRNAs, we performed qPCR for a panel of mature mouse miRNAs in human *Dicer* expressing iPSCs. As expected, these cells expressing human *Dicer* had comparable levels of mature miRNAs to that of wild-type ESCs (Fig 3H).

Our results indicate that miRNAs are essential for reprogramming since *Dicer*-null MEFs could not give rise to induced stem cell-like cells. Human *Dicer* rescued the ability to generate mature miRNAs in *Dicer* ^Δ/Δ^ MEFs, and restored their reprogramming potential. The dramatic proliferation delay in *Dicer*-null MEF likely contributes to inhibiting cellular reprogramming as it has been demonstrated that an accelerated kinetics of iPSC formation is directly proportional to the increase in cell proliferation [38]. However, besides promoting proliferation, miRNAs likely have other functions that are essential for reprogramming since it is known that they regulate numerous genes and exert multiple cellular effects. Furthermore, we were able to reprogram *Dicer* ^Δ/Δ^ MEFs one day after Cre induction when the cells already had a significant growth delay (Fig 1E), suggesting that the impaired proliferation is not the only variable in preventing reprogramming. Identifying specific miRNAs that enable reprogramming would give clues about their mechanism of action. Although the mechanism of action still needs clarification, our results indicate for the first time that miRNAs are indispensable for dedifferentiation reprogramming.

## Materials and Methods

### Ethics Statement

Animals were handled according to relevant national and international guidelines under the protocol number 2010N000120, approved by the Massachusetts General Hospital’s Subcommittee on Research Animal Care. The committee approved the experiments conducted in this study.

### Conditional *Dicer* Knockout Mice and Cell Culture

MEFs were prepared from E13.5 wild-type, *Dicer* ^f/+^, and *Dicer* ^f/f^ embryos and cultured in DMEM containing 10% fetal bovine serum (FBS), 2 mM L-glutamine, 1× nonessential amino acids and 0.1 mM 2-mercaptoethanol (Invitrogen). A germline-competent mouse ESC line (W4) and mouse iPSC lines were cultured on irradiated MEFs in serum-containing ESC medium, DMEM with 15% FBS, 2 mM L-glutamine, 1× nonessential amino acids, 0.1 mM 2-mercaptoethanol, and 1,000 U/ml leukemia inhibitory factor (Chemicon). To remove functional Dicer, MEFs were treated with Adeno-Cre virus (University of Iowa; Iowa City, IA), added at a multiplicity of infection (MOI) of ∼100 and performed further analysis.

### Reprogramming MEFs Using Transposon Vectors

MEFs were plated on six-well plates (5×10^5^ cells per well) 1 day before transfection. The next day (day 0), 2 µg of pCMV-mPBase34 and piggyBac transposon were transfected using Lipofectamine 2000 (Invitrogen) according to the manufacturer’s instructions. On day 1, transfected MEFs were trypsinized and replated onto feeder layers. On day 2, ESC medium was added. The medium was refreshed every other day. On day 7, medium was changed to serum free ESC medium, which contains 15% Knockout serum replacement (Invitrogen). Medium was refreshed every other day. On day 14, colonies were either stained using the alkaline phosphatase detection kit (Chemicon) and counted, or picked and further expanded.

### Reprogramming MEFs Using Lentiviral Transduction

The lentiviral constructs Stemcca OSKM and the tet-activator Fuw-m2RTTA (Addgene) were used to ectopically induce reprogramming genes. To generate viral particles 5.8×10^6^ 293T cells per 10 cm dish were transfected with respective vector and the packaging plasmids psPAX2 (Addgene) and pMS2.G (Addgene) in a ratio of 2∶1:1. 48 hours after transfection the supernatant comprising the viruses was collected, mixed in a ratio of 2 (Stemcca) : 1 (m2RTTA) and filtered through a 0.45 µm cellulose acetate filter. Finally, polybrene (Millipore) was added at a final concentration of 4 µg/ml to increase infection efficiencies. One day before transduction, MEFs were passaged onto six well plates to reach a density of 60–80% on the day of transduction (1–1.2×10^5^ cells/well). For transduction, the culture medium was removed, and new medium containing freshly produced virus suspension was added to cover the surface of the wells (800 µl/well). After 4 hour incubation at 37°C, 5% CO_2_, additional medium was added (2 ml total), and the cells were incubated overnight. The next day, the virus-containing medium was replaced by fresh culture medium. Medium was changed every day.

### Cell Proliferation Assay

Cell proliferation was performed using cell proliferation assay kit (Promega) according to the manufacturer’s instructions. After plating MEFs, cells were treated with Adeno-Cre virus and analyzed 1, 3 and 5 days after infection. At the indicated time points, medium was replaced with MTS media and incubated at 37°C for 3 hours. Absorbance was recorded at 490 nm.

### RT-PCR and Western Blot Analysis

Total RNA was extracted by using TriZol reagent (Invitrogen). After tailing, one microgram of total RNA was reverse-transcribed using an oligo(dT) adaptor primer by SuperScriptII (Invitrogen) according to the manufacturer’s instructions [39]. Quantitative RT-PCR was performed using Platinum SYBR Green qPCR superMix (Invitrogen) on the CFX96 Real-Time System (Bio-Rad). Serial dilutions of each RT-PCR product were used to generate a standard curve. Expression of individual transcripts was normalized to Gapdh expression. Protein blots were analyzed using antibodies to Dicer (1∶1000, Abcam) and to β-actin (1∶2000, Abcam).

### Bisulfite Genomic Sequencing Assay

Genomic DNA was isolated and then treated for bisulfite sequencing with EpiTect Bisulfite Sequencing kit (Qiagen). The treated DNA was then used to amplify sequences of interest. The resulting fragments were cloned using the TOPO TA Cloning Kit (Invitrogen) and sequenced with promoter fragment amplification primers for Oct4 (forward; GGTTTTTTAGAGGATGGTTGAGTG, reverse; TCCAACCCTACTAACCCATCACC) and Nanog (forward; GATTTTGTAGGTGGGATTAATTGTGAATTT, reverse; ACCAAAAAAACCCACACTCATATCAATATA) [34].

### Immunocytochemistry and Immunofluorescence Assay

Alkaline phosphatase staining was performed according to the manufacturer’s instructions using the Alkaline Phosphatase Detection Kit (Vector Lab). For immunofluorescence assay, cells were fixed in 4% paraformaldehyde for 15 minutes at room temperature (RT) and washed with PBS. They were then incubated in blocking buffer (0.3% Triton X-100, 10% normal goat serum in PBS) for 30 minutes at RT, and incubated with primary antibody overnight at 4°C in blocking buffer. Afterward, cells were washed with PBS and incubated with secondary antibody in blocking buffer for 45–60 min at RT. Primary antibodies were mouse anti-Oct4 (1∶400, Abcam), mouse anti-SSEA1 (1∶400, Developmental Studies Hybridoma Bank at the University of Iowa), and rabbit anti-Nanog (1∶500, Abcam). Secondary antibodies were Alexa Fluor 488, 555 donkey anti-mouse or rabbit IgG (1∶500, Invitrogen). Nuclei were detected by DAPI (Sigma-Aldrich) staining.

### Teratoma Formation

Approximately 1×10^6^ *hDicer* rescued *Dicer* ^Δ/Δ^ iPSCs, stem cell-like cells (*Dicer* ^Δ/Δ^), and control iPSCs were injected subcutaneously into dorsal flanks of recipient SCID mice. Tumors were isolated 4–6 weeks later and subjected to histological analysis.

## Supporting Information

Table S1 **Reprogramming efficiencies of various MEFs.** The overall reprogramming efficiencies were between 0.1% and 0.5% except for *Dicer* ^Δ/Δ^ MEFs which could not be reprogrammed into induced stem cell-like cells when either 4 TFs or 5 TFs were transduced 6 days after induction with Cre (*Dicer* ^Δ/Δ^-6dpi).
(TIF)
