# Supplementary figures and images for: MicroRNAs Are Indispensable for Reprogramming Mouse Embryonic Fibroblasts into Induced Stem Cell-Like Cells
Source: PLoS One. 2012 Jun 21;7(6):e39239. doi: 10.1371/journal.pone.0039239 (PMC3380844; doi:10.1371/journal.pone.0039239)

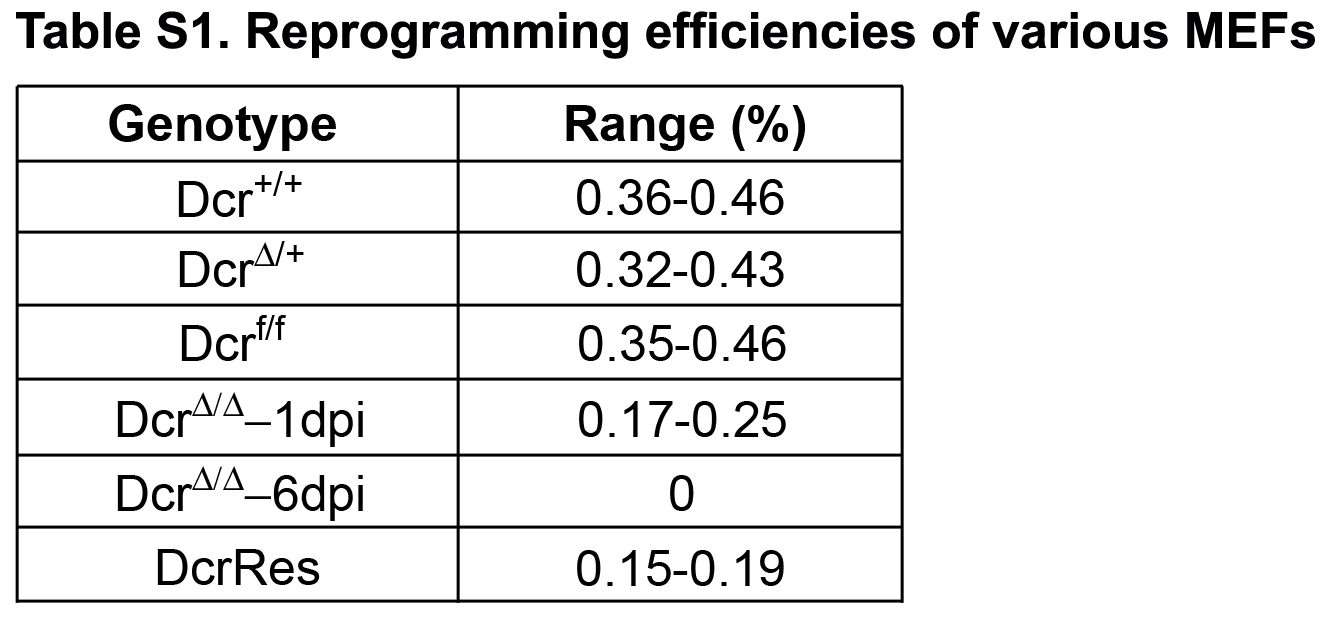

Supplement: Table S1 — Reprogramming efficiencies of various MEFs. The overall reprogramming efficiencies were between 0.1% and 0.5% except for Dicer Δ/Δ MEFs which could not be reprogrammed into induced stem cell-like cells when either 4 TFs or 5 TFs were transduced 6 days after induction with Cre (Dicer Δ/Δ-6dpi). (TIF) [file pone.0039239.s001.tif]
